# On Vascular Lesions of the Thyroid Gland with Emphasis on Intrathyroidal Hemangioma: Clinicopathologic Characterization of Two Cases and Review of the Literature

**DOI:** 10.1007/s12105-024-01722-6

**Published:** 2024-10-18

**Authors:** William W. MacDonald, Paul E. Wakely, Prokopios P. Argyris

**Affiliations:** 1 Department of Pathology, The Ohio State University Wexner Medical Center, James Cancer Hospital and Solove Research Institute, Columbus, OH USA; 2 Division of Oral and Maxillofacial Pathology, The Ohio State University College of Dentistry, Postle Hall, Room 2191 305 W. 12th Ave, Columbus, OH 43210 USA

**Keywords:** Thyroid Hemangioma, Thyroid Angiosarcoma, Masson Tumor, Intravascular Papillary Endothelial Hyperplasia, Thyroid Arteriovenous Malformation, Thyroid Vascular Neoplasm, Thyroid Epithelioid Hemangioendothelioma, Thyroid Kaposi Sarcoma

## Abstract

Mesenchymal neoplasms of the thyroid gland are exceptionally rare accounting for less than 0.5% of all intrathyroidal tumors with hemangiomas comprising merely 6% of them. The clinicopathologic characteristics of two additional examples of thyroid hemangioma together with a thorough review of the pertinent literature are presented. A 62-year-old man and an 18-year-old woman presented with asymptomatic, soft-to-palpation, mobile nodules of the right thyroid lobe classified as TI-RADS 5 and TI-RADS 4, respectively, on ultrasound imaging. Microscopically, lesions featured a circumscribed, unencapsulated, lobular proliferation of variably-sized, congested, vascular channels lined by a single layer of flattened, cytologically bland endothelial cells, together with interspersed residual follicles. Vascular endothelial cells were strongly positive for CD31, CD34 and ERG, and negative for pancytokeratin AE1/AE3, TTF1, and PAX8. A diagnosis of cavernous hemangioma was rendered in the clinical setting of Hashimoto thyroiditis and follicular adenoma, respectively. Following inclusion of the current cases, a total of 53 intrathyroidal hemangiomas were identified in the literature with a patient mean age of 48.9 years (range = 0.17-84) and a slight female predilection (F:M = 1.4:1). A proclivity for the right thyroid lobe (59.6%) was noted with the striking majority of cases exhibiting features of cavernous hemangioma (95.2%). Prognosis is favorable and surgical resection is considered curative. The occasionally alarming clinical presentation in conjunction with absence of pathognomonic imaging features and limited diagnostic accuracy of FNA cytopathology for such lesions renders surgical intervention necessary for definitive diagnosis of intrathyroidal hemangiomas and exclusion of other epithelial and non-epithelial pathologic entities.

## Introduction

Mesenchymal neoplasms of the thyroid gland are extremely rare comprising less than 0.5% of thyroid tumors and, when primary, they originate from the mesenchymal components of the thyroid, i.e., fibrous connective tissue stroma and lymphovascular channels [1, 2]. Secondary mesenchymal tumors occur more frequently and represent the sequelae of intrathyroidal metastasis or locoregional involvement by adjacent head and neck malignancies [2, 3]. The histopathologic landscape of thyroid mesenchymal neoplasms is markedly broad with over 30 different histologic subtypes currently reported [2, 4]. Among them, malignant neoplasms of vascular endothelial cell lineage, mainly angiosarcoma, predominate and account for 13% of all mesenchymal tumors [2] and 36.7% of intrathyroidal sarcomas, respectively [5].

Benign vascular tumors of the thyroid encompass a variety of lesions, including hemangioma, lymphangioma and vascular (arteriovenous) malformations, characterized by a proliferation of variably-sized, mature blood and/or lymphatic vessels lined by bland endothelial cells lacking evidence of pronounced cytologic atypia [4]. Intrathyroidal hemangiomas are exceptionally uncommon accounting for merely 6% of all mesenchymal neoplasms of the thyroid gland [2] and usually present clinically as solitary, asymptomatic and slow-progressing, cervical nodules [6, 7]. Occasionally, rapid growth due to hemorrhage and/or presence of calcifications may raise suspicion for thyroid carcinoma [4, 7, 8]. Etiologically, at least a portion of thyroid hemangiomas are postulated to represent nonneoplastic vascular anomalies or the result of fine needle aspiration biopsy (FNAB) – induced trauma [9]. Due to absence of pathognomonic clinical and/or imaging findings, a definitive diagnosis of intrathyroidal vascular tumors may be challenging prior to surgical intervention [10].

Herein, we present the clinicopathologic characteristics of two additional rare examples of intrathyroidal cavernous hemangioma, review the existing literature, and provide a thorough discussion on the histopathologic spectrum of vasoproliferative tumors affecting the thyroid gland.

## Methods and Materials

Publicly available databases including PubMed, Medline, Google Scholar and ResearchGate were searched for previously reported examples of thyroid hemangioma using the following keywords: “thyroid hemangioma”, “cavernous hemangioma of the thyroid”, “capillary thyroid hemangioma”, “venous thyroid hemangioma”, “thyroid vascular tumor”, and “thyroid mesenchymal tumor”. Well-documented, original articles in the form of single case reports or case series published during the period 1975–2024 were retrieved. Inclusion criteria were as follows: (i) adequate documentation of the diagnosis by means of imaging, histopathologic examination or FNA cytopathology, and (ii) availability of the full article or pertinent abstract in the English language, or (iii) non-English case reports included and discussed in English-written review studies. Thirty-nine original reports fulfilling the above criteria were identified leading to a total of *N* = 51 cases of intrathyroidal hemangioma [3, 6–42]. Available information regarding patient age and gender, as well as lesion size, laterality, histopathologic subtype and treatment modality was collected and tabulated (Table 1).

**Table 1:** Collective presentation of the demographic and clinicopathologic characteristics of all previously reported examples of intrathyroidal hemangioma including the two cases of the current study

Author (Year)	Cases	Age (years)	Gender	Size (cm)	Laterality	Hemangioma Subtype	Treatment
Pickleman et al. (1975)	1	56	M	7.5	Left	Cavernous	Lobectomy
Shamim (1978)	1	30	M	NA	Right	Cavernous	Resection, NOS
Queiroz et al. (1978)	3	29	F	6.5	Right	Cavernous	Resection, NOS
		33	M	9.0	Right	Cavernous	Resection, NOS
		54	M	9.0	Right	Cavernous	Resection, NOS
Hernández et al. (1979)	1	55	F	2.0	Right	Capillary	Resection, NOS
Ismaĭlov et al. (1981)	1	55	M	10	Right	Cavernous	Resection, NOS
Ishida et al. (1982)	3	57	F	8.0	Right	Cavernous	Resection, NOS
		4	M	1.8	Isthmus	Cavernous	Resection, NOS
		46	M	14	Right	Cavernous	Resection, NOS
Yokota et al. (1991)	1	64	F	7.2	Right	Cavernous	Resection, NOS
Pendse and Porwal (1998)	1	53	M	6.0	Right	Cavernous	Lobectomy
Clarke and Boppana (1998)	1	17	F	9.0	Left	Cavernous	Resection, NOS
Kumar et al. (2000)	1	53	M	4.0	Right	Cavernous	Resection, NOS
Ríos et al. (2001)	2	48	F	5.0	Left	Cavernous	Total thyroidectomy
		63	F	5.0	Left	Cavernous	Lobectomy
Suzuki (2004)	1	28	F	6.0	Left	Cavernous	Lobectomy
Kumamoto et al. (2005)	1	56	F	7.0	Right	Cavernous	Lobectomy
Senthilvel et al. (2005)	1	24	M	6.0	Right	NA; diagnosis by imaging only	NA
Kano et al. (2005)	1	21	M	5.5	Right	Cavernous	Lobectomy
Lee et al. (2007)	1	66	M	17	Left	Cavernous	Thyroidectomy
Ciralik et al. (2008)	1	64	M	7.0	Right	Cavernous	Resection, NOS
Datta et al. (2008)	1	25	M	4.9	Left	Cavernous	Resection, NOS
Sakai et al. (2009)	1	71	F	5.0	Left	Cavernous	Subtotal thyroidectomy
Michalopoulos et al. (2010)	1	78	M	4.0	Right	Cavernous	Total thyroidectomy
Maciel et al. (2011)	1	80	F	22	Left	Cavernous	Lobectomy
Gutzeit et al. (2011)	1	84	F	4.0	Left	Cavernous	Lobectomy
Jacobson et al. (2014)	1	0.25 (3 months)	F	3.1	Right	NA; diagnosis by imaging only	NA
Dasgupta et al. (2014)	1	38	M	6.0	Left	Cavernous	Lobectomy
Karanis et al. (2015)	1	45	F	6.0	Right	Cavernous	Lobectomy
Miao et al. (2017)	1	48	M	4.0	Right	Cavernous	Lobectomy
Liang et al. (2020)	1	0.17 (2 months)	F	3.0	Bilateral	Capillary	Lobectomy
Yang et al. (2020)	1	24	F	3.6	Left	Hemangioma, NOS	Resection, NOS
Bains et al. (2020)	1	77	F	1.2	Right	Cavernous	Lobectomy
Masuom et al. (2021)	1	63	M	7.0	Right	Cavernous	Total thyroidectomy
Cristel et al. (2021)	1	56	F	4.0	Right	Hemangioma, NOS	Total thyroidectomy
Díaz-García et al. (2022)	1	51	F	5.5	Right	Cavernous	Lobectomy
Mukhuba and Bhuiyan (2022)	1	51	M	7.5	Left	Cavernous	Lobectomy
Seuferling et al. (2022)	1	62	F	1.5	Right	Cavernous	Resection, NOS
Zhang et al. (2022)	2	75	F	1.0	NA	Hemangioma, NOS	Resection, NOS
		49	M	1.0	NA	Hemangioma, NOS	Resection, NOS
Berdica et al. (2023)	4	73	F	6.0	Left	Cavernous	Total thyroidectomy
		61	F	5.5	Right	Cavernous	Total thyroidectomy
		32	F	4.0	Left	Cavernous	Total thyroidectomy
		38	F	3.0	Left	Cavernous	Total thyroidectomy
Saoud et al. (2023)	4	44	F	2.5	NA	NA; diagnosis by cytology only	Observation
		41	F	2.1	NA	Hemangioma, NOS	Lobectomy
		75	F	10	NA	Hemangioma, NOS	Total thyroidectomy
		84	M	3.3	NA	NA; diagnosis by cytology only	Observation
Al-Maghrabi et al. (2024)	1	76	F	7.5	Right	Hemangioma, NOS	Resection, NOS
Coetzee et al. (2024)	1	36	F	0.8	Left	Cavernous	Lobectomy
Current study	2	62	M	2.6	Right	Cavernous	Lobectomy
		18	F	NA	Right	Cavernous	Lobectomy, isthmusectomy
**Total Number of Cases**	**53**	**Mean = 48.9;** **Range = 0.17–84**	**31 F:22 M;** **F: M = 1.4:1**	**Mean = 5.8;** **Range = 0.8–22**			

NA; not available, NOS; not otherwise specified

## Case Presentation

### Case 1

A 62-year-old man with a familial history of hypothyroidism chronically managed with levothyroxine presented with painless fullness of the right thyroid gland. Voice hoarseness, cervical lymphadenopathy, or other compressive symptoms were not appreciated. The patient denied previous cervical surgeries or trauma. Upon physical examination, an approximately 3 cm, soft-to-palpation nodule of the right thyroid lobe was identified. Laboratory tests disclosed a supratherapeutic thyroid-stimulating hormone with normal T4 levels. Ultrasound imaging revealed a 2.6 cm nodule with complex, cystic and solid, architecture involving the inferior aspect of the right thyroid lobe, classified as TI-RADS score 5. FNAB was performed and interpreted as concerning for papillary thyroid carcinoma. Subsequently, the patient underwent right thyroid lobectomy together with level VI lymph node dissection.

**Fig. 1:**
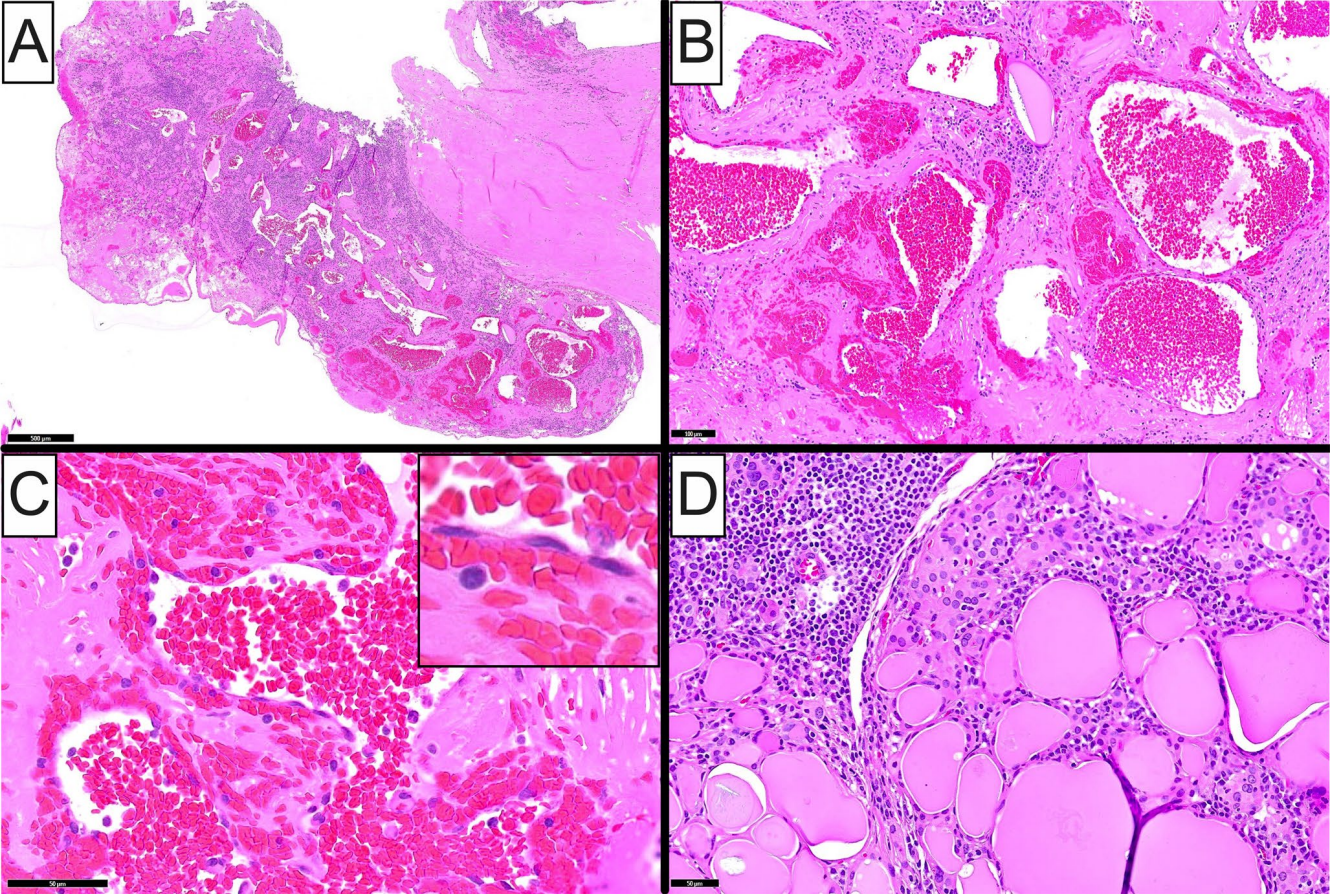
Histopathologic features of intrathyroidal cavernous hemangioma (Case 1). (**A**) Low-power photomicrograph of the resected specimen depicting an intraluminal, lobular, vascular proliferation in association with dense fibrocollagenous connective tissue. (**B**) Medium-power photomicrograph showing numerous, congested, occasionally anastomosing, vascular channels of variable size and shape, immersed in a fibrous, markedly hemorrhagic stroma. (**C**) High-power photomicrograph showing thin-walled vascular spaces lined by single layer of flattened, bland, endothelial cells; (**inset**) endothelial cells lack cytologic atypia and mitotic activity. (**D**) Medium-power photomicrograph of the adjacent thyroid parenchyma displaying marked chronic lymphocytic infiltrates consistent with Hashimoto thyroiditis

**Fig. 2:**
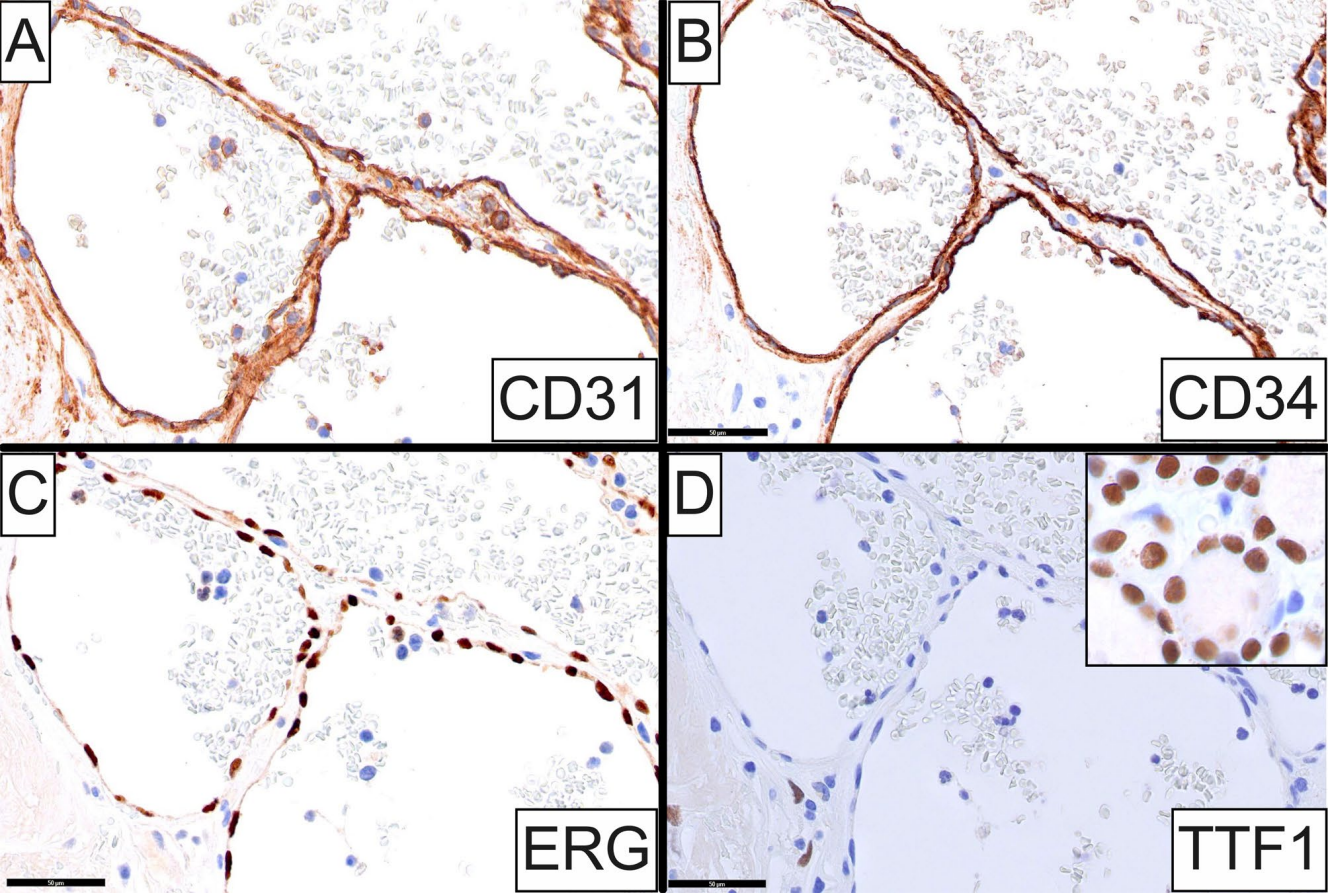
Immunophenotypic characteristics of intrathyroidal cavernous hemangioma (Case 1). Endothelial cells lining lesional vascular channels exhibit strong positivity for CD31 (**A**), CD34 (**B**), and ERG (**C**), while they are uniformly negative for TTF1 (**D**) which is strongly expressed in adjacent thyroid follicular cells (**inset**)

Gross examination showed a well-defined, multiloculated, cystic nodule filled with serosanguinous fluid abutting the external surface of the inferior thyroid pole. On cut surface, a 0.9 cm, hemorrhagic, papillary, luminal excrescence was noted stemming from the cystic wall. Microscopically, the lesion featured a central cystic cavity enveloped by dense fibrocollagenous connective tissue. A lobular proliferation of variably-sized, congested, occasionally anastomosing vascular channels was observed involving the cystic lumen as well as the fibrous cyst wall, along with prominent interstitial hemorrhage (Fig. 1A and **B**). The blood vessels featured a single layer of flattened endothelial cells lacking cytologic atypia and mitotic activity (Fig. 1C and C inset). The adjacent thyroid parenchyma exhibited marked chronic lymphocytic infiltrates consistent with Hashimoto thyroiditis (Fig. 1D). By immunohistochemistry, the vascular endothelial cells were strongly positive for CD31 (Fig. 2A), CD34 (Fig. 2B) and ERG (Fig. 2C), and negative for pancytokeratin AE1/AE3, TTF1, and PAX8 (Fig. 2D). A diagnosis of intrathyroidal cavernous hemangioma was rendered. No evidence of papillary thyroid carcinoma was appreciated in the residual thyroid tissue, while all intrathyroidal and level VI lymph nodes examined showed unremarkable histopathologic findings.

### Case 2

An 18-year-old woman with a history of anemia presented with hair loss, increased acne, and unilateral neck fullness. No dysphagia or shortness of breath was reported and thyroid function laboratory results were within normal limits. Clinically, an asymptomatic, soft-to-palpation, mobile nodule of the right thyroid lobe was identified. Ultrasound imaging disclosed a 4.6 cm, hypoechoic, solid mass scored as TI-RADS 4. An incisional biopsy was performed and showed hyperplastic follicular tissue with the patient undergoing right lobectomy and isthmusectomy a month later.

**Fig. 3:**
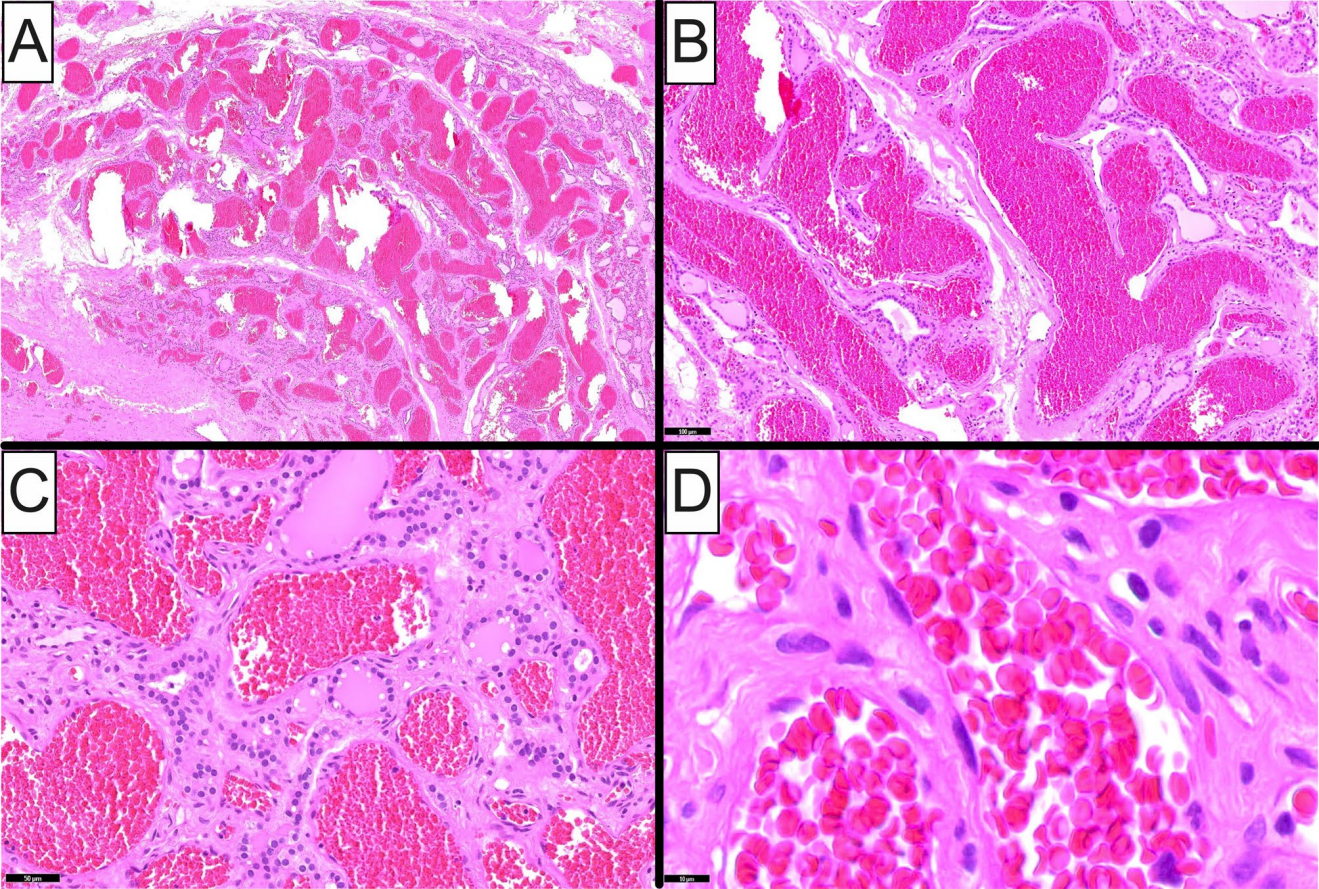
Histopathologic features of intrathyroidal cavernous hemangioma (Case 2). (**A**) Low-power photomicrograph depicting an unencapsulated but circumscribed, lobular proliferation of numerous, thin-walled, vascular spaces. (**B**) Low-power photomicrograph showing numerous, congested, varying-in-caliber vascular channels. (**C**) Medium-power photomicrograph showing entrapped residual thyroid follicles among the cavernous vascular spaces. (**D**) High-power photomicrograph highlighting the monolayer of flattened, cytologically bland, endothelial cells lining the lesional vessels

Gross examination of the excisional specimen revealed a well-circumscribed, 3.5 cm, solid nodule located in the mid-to-lower aspect of the right thyroid lobe. Marked hemorrhage occupying ~ 50% of the cut surface of the specimen was noted upon sectioning. Microscopically, the lesion comprised an unencapsulated but circumscribed, lobular proliferation of numerous, thin-walled, vascular spaces (Fig. 3A and **B**), in the vicinity of a follicular adenoma. The congested, varying-in-caliber, vascular channels (Fig. 3B and **C**) were lined by a monolayer of flattened, bland, endothelial cells (Fig. 3D). Entrapped residual follicles were noted among the cavernous vascular spaces (Fig. 3C). A diagnosis of cavernous hemangioma of the thyroid in association with a follicular adenoma was made.

## Discussion

Primary thyroid neoplasms are traditionally categorized into epithelial and non-epithelial, with the latter further subclassified according to their cell lineage into lymphoid and mesenchymal [1–3]. Owing to their rarity, robust epidemiologic data regarding the incidence and prevalence of intrathyroidal mesenchymal neoplasms are currently sparse with such tumors comprising just a minute fraction of all tumors involving the thyroid gland [1, 2] and only ~ 6% of them encompassing hemangiomas [2]. A thorough review of the literature revealed 51 previously reported examples of intrathyroidal hemangioma summarized in Table 1 [3, 6–42]. When the two current cases are also considered (*N* = 53), hemangiomas of the thyroid exhibit a slight female predilection (31 F:22 M; F:M ratio = 1.4:1) with a mean age of 48.9 years and a markedly broad age range (0.17–84 years). A proclivity for the right thyroid lobe is noted (59.6%), followed by the left lobe (36.1%), and one case each (2.1%) occurring in the isthmus or bilaterally. Cavernous is largely the most common histopathologic subtype of intrathyroidal hemangioma (95.2%), while less commonly the capillary type (4.8%) may be seen. Clinically, lesions present as slow-growing, painless, anterior neck swellings or masses of variable size (range = 0.8–22 cm; mean = 5.8 cm). Compressive symptoms including tracheal deviation, dyspnea, voice hoarseness, and night sweats have also been reported [8, 11, 23, 33, 36, 38]. Although most cases occur in adulthood, sparse examples of congenital intrathyroidal hemangiomas have also been reported [32] and are postulated to derive from errors in vasculogenesis [3, 9]. Intrathyroidal hemangiomas may develop *de novo* or in the clinical setting of other inflammatory or neoplastic processes including Hashimoto thyroiditis and follicular adenoma [41], as reported here. Rarely, the tautochronous development of thyroid hemangioma and papillary thyroid carcinoma may also occur [36]. The frank neoplastic nature of intrathyroidal hemangiomas has been frequently refuted with a substantial body of evidence supporting that many of the so-called “primary hemangiomas” most likely represent nonneoplastic vascular malformations [4, 41].

Notwithstanding the clinical utility of ultrasound guided FNAB in the diagnosis of thyroid epithelial neoplasms, diagnosis of intrathyroidal hemangiomas solely on cytology smears is exceptionally challenging since such lesions are often acellular or scantly cellular and demonstrate rare spindle or polygonal endothelial cells with bland ovoid nuclei, fine chromatin and inconspicuous nucleoli in a background of abundant blood [3, 38]. Assuring collection of adequate material for a cell block in conjunction with ancillary immunohistochemical studies could enhance the diagnostic accuracy of FNAB [3]. Notably, traumatic events such as FNAB may lead to the formation of local hematoma with subsequent, abnormal, fibroblastic and vascular proliferation mimicking intrathyroidal cavernous hemangioma [3, 9, 43]. The incidence of post-FNAB thyroid vascular proliferations ranges between 2.7% [44] to 8.1% [45] and even approximates 45% of cases in certain studies [46].

Ultrasonography and contrast-enhanced CT imaging are routinely utilized preoperatively for assessment of thyroid nodules. However, adding to the level of diagnostic complexity, intrathyroidal hemangiomas lack definitive and specific imaging features [3, 8]. Indeed, diagnosis was based exclusively on imaging findings in only 2 of 49 previously reported thyroid hemangiomas [23, 32]. Various other imaging techniques, e.g., MRI, contrast-enhanced ultrasound, digital subtraction angiography and single-photon emission CT have been proposed to enhance preoperative diagnostic accuracy for hemangiomas of the thyroid [34]. Be that as it may, surgical resection, i.e., lobectomy or total thyroidectomy, and histopathologic examination remain standard of care for the diagnosis and management of intrathyroidal hemangioma [8, 37, 38, 40, 41].

As mentioned above, distinction between intrathyroidal hemangioma and vascular malformations involving the thyroid gland may be proven impossible based on histomorphology alone. Vascular malformations derive from errors in development and morphogenesis and primarily affect the skin of the maxillofacial region with involvement of visceral organs and bones being less common [47, 48]. Arterial, venous, capillary, and lymphatic malformations, as well as combinations of them, have been reported [47, 48]. Arteriovenous malformations of the thyroid are considered rare and, similar to extra-thyroidal anatomic sites, they comprise a mixed population of variably-sized veins, arteries, and occasionally lymphatics, with the venous component typically predominating [4, 49]. Thrombosis and phlebolith formation may be present [4]. Reports on arteriovenous malformations of the thyroid are sparse [49–52] and infrequently an association with underlying systemic conditions such as Wyburn-Mason syndrome [53] and hereditary hemorrhagic telangiectasia [54] has been reported.

In addition to hemangioma and vascular malformations, intravascular papillary endothelial hyperplasia (IPEH), i.e., Masson tumor, may also involve the thyroid, albeit such an event is uncommon [55–57]. Most examples of IPEH and PEH vascular proliferations are postulated to occur due to neovasculogenesis and recanalization in the setting of extensive FNAB-induced blood extravasation [45, 58, 59]. Furthermore, IPEH parenchymal changes may be encountered in long-standing and infarcted goiter nodules undergoing regressive phenomena, including edema, fibrosis, calcification and hemorrhage without a previous FNAB [60, 61]. IPEH lesions are, overall, well-circumscribed, lobulated, occasionally enveloped by a fibrous pseudocapsule and may exhibit remarkable size [57, 61]. On cut surface, a variably cystic architecture is identified in association with pronounced hemorrhage and a solid component comprising anastomosing hyperplastic blood vessels, fibrin, granulation tissue and collagen [61]. Numerous papillary fronds are noted that are lined by a single layer of plump endothelial cells and supported by delicate fibrous cores protruding into the vascular spaces. Microthrombi are often present. Although modest nuclear atypia, e.g., nucleomegaly with coarse chromatin distribution, and scattered mitotic figures may be seen in IPEH, frank histopathologic characteristics concerning for malignancy such as geographic necrosis, extensive high-grade cytologic atypia with increased atypical mitoses, and “piling-up” of endothelial cells are absent or unusual [61]. Of note, focal or florid PEH may develop within or in the vicinity of the capsule of thyroid carcinomas [56, 61, 62]. Interestingly, the endothelial cells lining the papillae of pericapsular PEH associated with thyroid neoplasms are strongly positive for D2-40 confirming their lymphatic lineage and supporting a possible etiologic relationship with lymphangiogenic factors secreted by neoplastic follicular cells [62].

**Fig. 4:**
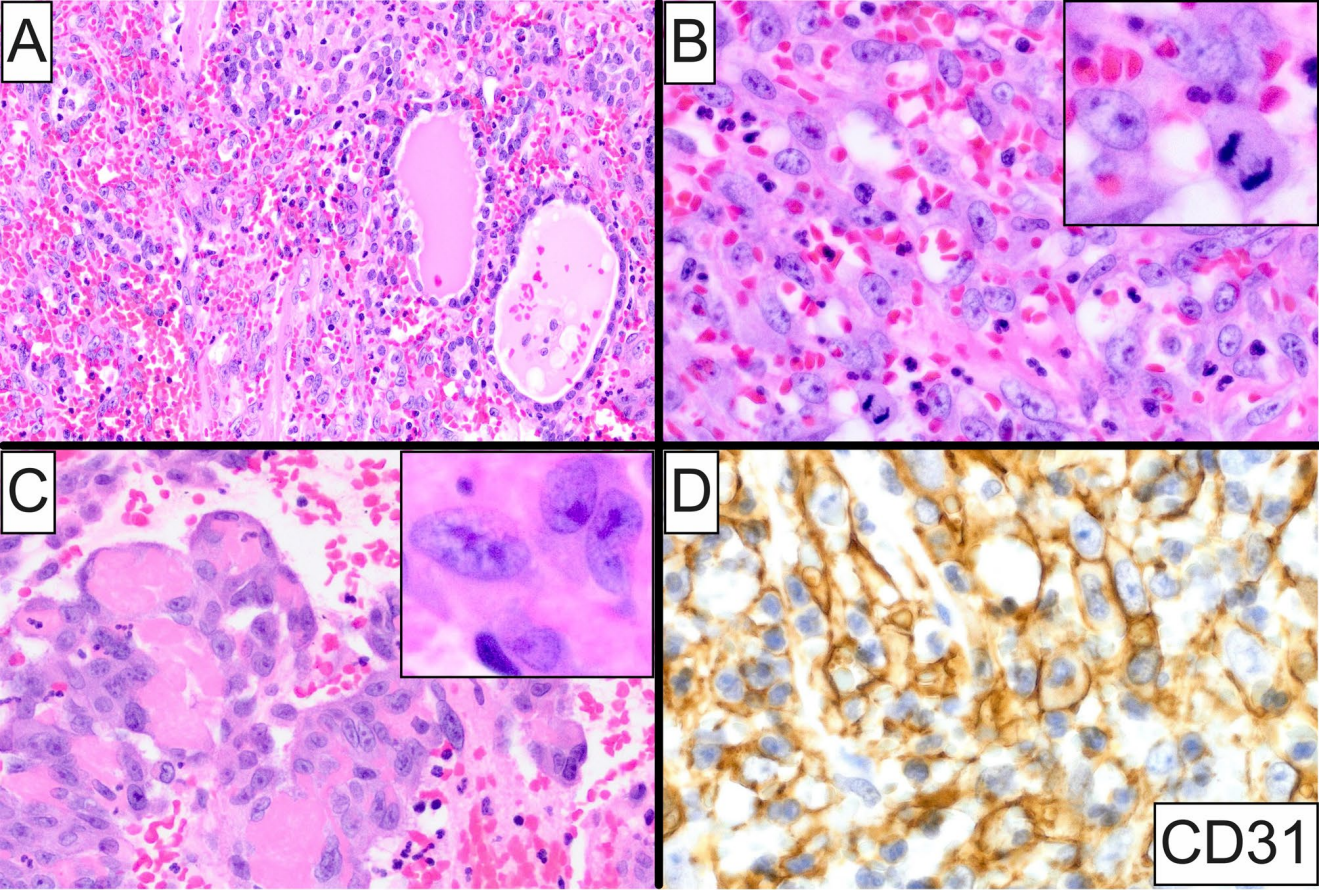
Histopathologic and immunohistochemical characteristics of primary angiosarcoma of the thyroid. (**A**) Low-power photomicrograph showing a highly atypical vasoformative neoplasm that exhibits a diffusely infiltrative growth pattern with destruction of adjacent follicular epithelium. Pronounced interstitial hemorrhage is noted. (**B**) High-power photomicrograph depicting dilated and anastomosing vascular spaces lined by overtly malignant, epithelioid and spindle cells featuring enlarged, oval, vesicular nuclei with coarse chromatin, one or more macronucleoli, and abundant eosinophilic cytoplasm; (**inset**) mitotic figures are readily identifiable. (**C**) High-power photomicrograph showing sarcoma cells tufting within a vascular lumen; (**inset**) malignant epithelioid cells featuring nucleomegaly, acidophilic macronucleoli and rich eosinophilic cytoplasm. (**D**) Lesional cells show strong and diffuse positivity for CD31

Primary thyroid sarcomas represent rare neoplasms accounting for less than 1% of all sarcoma diagnoses [63] and 55% of all intrathyroidal mesenchymal tumors [2]. Angiosarcoma and epithelioid hemangioendothelioma predominate among other histologic subtypes collectively comprising 13% of all thyroid mesenchymal neoplasms and 23.4 − 36.7% of all mesenchymal malignancies, respectively [2, 5]. Angiosarcoma accounts for approximately 15 − 20% of all thyroid sarcomas [5, 64] and shows a predilection for women in their sixth to eighth decade of life [3, 64]. A higher incidence is noted in alpine countries of central Europe, where there is a known deficiency of dietary iodine, although, cases have also been documented in regions with adequate iodine intake [3, 64–66]. A rapidly enlarging neck mass over a few months period with associated compression symptoms, i.e., dyspnea, hoarseness and dysphagia, is the most common clinical presentation [3, 63, 64, 67–69]. Paraneoplastic hypercalcemia, transient increase of blood sugar or leukocytosis are not uncommon [4, 64]. Microscopically, primary thyroid angiosarcoma exhibits a diffusely infiltrative growth pattern with destruction of adjacent follicular epithelium (Fig. 4A) and frequent extrathyroidal extension. Dilated, ramifying and anastomosing vascular spaces lined by atypical or overtly malignant endothelial cells are usually seen immersed in a markedly hemorrhagic fibrous background (Fig. 4A-C). Occasionally, the vasoformative architecture may be obscured by a solid cellular proliferation composed of chiefly epithelioid and/or spindle-shaped cells featuring enlarged, oval, vesicular nuclei with well-defined nuclear membrane, coarse chromatin with one or more macronucleoli, and abundant eosinophilic or amphophilic cytoplasm (Fig. 4B, C and insets). Mitotic figures, including atypical forms, are readily identifiable (Fig. 4B inset). Vascular tufting (Fig. 4C) and intracytoplasmic microlumina with or without the presence of erythrocytes may be seen. The main histopathologic differential diagnosis of primary angiosarcoma of the thyroid includes anaplastic thyroid carcinoma with angiosarcomatous (angiomatoid) features [61, 64, 70]. By immunohistochemistry, thyroid angiosarcomas are strongly and diffusely positive for pan-endothelial markers, i.e., CD31 (Fig. 4D), CD34, FLI-1 and ERG, but lack expression of thyroid-related immunomarkers including thyroglobulin, TTF1 and PAX8 [4, 61, 64, 67, 70]. Notably, strong and diffuse staining pattern against low-molecular weight cytokeratins can be seen in up to 50% of primary thyroid angiosarcomas, especially in those with epithelioid histomorphology [4, 70–72], posing an important diagnostic pitfall. Conversely, anaplastic thyroid carcinomas retain cytokeratin expression in approximately 70% of cases [73] with 54 − 70% of them also exhibiting strong PAX8 staining [73, 74], whereas reactivity for vascular markers is only focal and observed in a minor subset of tumors. Finally, in contrast to intrathyroidal angiosarcomas that, overall, harbor a low mutational burden, anaplastic thyroid carcinomas display a complex genomic landscape with frequent *TP53* (40–80%) and *TERT* promoter (30–75%) aberrations, together with *RAS* and *BRAF V600E* (10–50% each) mutations among others [73, 75].

Besides angiosarcoma, the histopathologic repertoire of malignant vascular neoplasms that may involve the thyroid gland includes epithelioid hemangioendothelioma and Kaposi sarcoma accounting for 16.3% and 1.4% of all primary thyroid mesenchymal malignancies, respectively [5]. The exact prevalence of *bona fide* intrathyroidal epithelioid hemangioendothelioma is unclear since the term “malignant hemangioendothelioma” has been variably utilized in the literature to often include lesions better classified as thyroid angiosarcoma [76]. Only five previous examples of thyroid epithelioid hemangioendothelioma have been reported in the English literature with all cases affecting women (age median = 44 years; range = 35–74 years) [42, 76–79]. A rapidly growing cervical mass (size range: 2.1–8.0 cm) in association with local neck discomfort, progressive voice hoarseness, dysphagia and weight loss comprised frequent clinical findings [76, 77, 79]. Similar to extrathyroidal epithelioid hemangioendotheliomas, intrathyroidal lesions demonstrated a diffusely infiltrative population of spindled to epithelioid cells organized in short strands, cords or solid nests embedded in a myxohyaline, chondroid-like stroma [76–79]. Tumor cells featured round to ovoid nuclei with often vesicular chromatin and inconspicuous nucleoli, abundant glassy eosinophilic cytoplasm and well-defined cell membrane borders. Intracytoplasmic vacuoles imparting the appearance of “blister cells” were occasionally seen [76, 77, 79]. Overall, nuclear pleomorphism and cytologic atypia were minimal, while mitotic activity was limited or absent [76–79]. Lymph node metastasis together with marked extrathyroidal extension and perineural invasion have also been documented [76]. As anticipated, thyroid epithelioid hemangioendotheliomas were strongly positive for CD31, CD34 and ERG [76–79], focally immunoreactive for cytokeratins and negative for thyroglobulin and TTF1 [78]. Furthermore, molecular analyses performed in 2 cases disclosed the presence of a t(1;3)(p36.3;q25) resulting in the *WWTR1::CAMTA1* fusion [42, 76].

Kaposi sarcoma of the thyroid gland is extremely uncommon with only scattered cases reported and almost exclusively in the clinical setting of HIV/AIDS [80–83]. A single occurrence in an immunocompetent woman has been documented [84]. Clinically, lesions presented as painless palpable thyroid nodule(s) or mass in association with hypothyroidism [81] or cervical lymphadenopathy [82]. Notably, an uncommon type of intravascular endothelial proliferation involving the capsular vessels of follicular-derived thyroid carcinomas has been described bearing overt histopathologic resemblance to Kaposi sarcoma [85]. Such Kaposi-like, possibly reactive in nature, endothelial processes appear to be unrelated to FNAB and comprise a spindle cell population featuring plump, cytologically bland nuclei lacking mitotic activity, with focal nesting and occasional interspersed erythrocytes. Immunostaining against HHV8 is helpful in discerning Kaposi sarcoma from its histologic mimics.

In conclusion, we present the clinicopathologic characteristics of two additional examples of intrathyroidal cavernous hemangioma, enriching the existing literature regarding this rare vascular tumor. Overall, thyroid hemangiomas show a slight preponderance for women and an incidence peak in the 5th decade of life. The occasionally alarming clinical presentation in conjunction with absence of pathognomonic imaging features and limited diagnostic accuracy of FNA cytopathology for such lesions renders surgical intervention necessary for definitive diagnosis of intrathyroidal hemangiomas and exclusion of other epithelial and non-epithelial pathologic entities. Prognosis is favorable and surgical resection is considered curative.

## Data Availability

No datasets were generated or analysed during the current study.
